# The effect of acetaminophen (four grams a day for three consecutive days) on hepatic tests in alcoholic patients – a multicenter randomized study

**DOI:** 10.1186/1741-7015-5-13

**Published:** 2007-05-30

**Authors:** EK Kuffner, JL Green, GM Bogdan, PC Knox, RB Palmer, K Heard, JT Slattery, RC Dart

**Affiliations:** 1 Rocky Mountain Poison & Drug Center, Denver Health, Denver, CO, USA; 2 Recovery Centers of King County, Seattle, WA, USA; 3 University of Washington, Seattle, WA, USA

## Abstract

**Background:**

Hepatic failure has been associated with reported therapeutic use of acetaminophen by alcoholic patients. The highest risk period for alcoholic patients is immediately after discontinuation of alcohol intake. This period exhibits the largest increase in CYP2E1 induction and lowest glutathione levels. Our hypothesis was that common liver tests would be unaffected by administration of the maximum recommended daily dosage of acetaminophen for 3 consecutive days to newly-abstinent alcoholic subjects.

**Methods:**

Adult alcoholic subjects entering two alcohol detoxification centers were enrolled in a prospective double-blind, randomized, placebo-controlled trial. Subjects were randomized to acetaminophen, 4 g/day, or placebo for 3 consecutive days. The study had 95% probability of detecting a 15 IU/L difference in serum ALT.

**Results:**

A total of 443 subjects were enrolled: 308 (258 completed) received acetaminophen and 135 subjects (114 completed) received placebo. Study groups did not differ in demographics, alcohol consumption, nutritional status or baseline laboratory assessments. The peak mean ALT activity was 57 ± 45 IU/L and 55 ± 48 IU/L in the acetaminophen and placebo groups, respectively. Subgroup analyses for subjects presenting with an elevated ALT, subjects fulfilling a diagnosis of alcoholic hepatitis and subjects attaining a peak ALT greater than 200 IU/L showed no statistical difference between the acetaminophen and control groups. The one participant developing an increased international normalized ratio was in the placebo group.

**Conclusion:**

Alcoholic patients treated with the maximum recommended daily dose of acetaminophen for 3 consecutive days did not develop increases in serum transaminase or other measures of liver injury. Treatment of pain or fever for 3 days with acetaminophen appears safe in newly-abstinent alcoholic patients, such as those presenting for acute medical care.

## Background

Acetaminophen (paracetamol) is a safe and effective over-the-counter (OTC) analgesic and antipyretic that has been used clinically for over 50 years. It is widely considered an appropriate first-line treatment for patients with fever, as well as for a variety of conditions causing mild to moderate pain.

Acetaminophen is used by over 50 million adults in the United States each week [1]. Reports of hepatic failure and death following the intended therapeutic use of acetaminophen by patients who consume alcohol have been published. Some practitioners recommend that the maximum dose of acetaminophen be lowered from the current dose of 4 g/day or that acetaminophen be avoided completely in alcoholic patients. As acetaminophen is used without injury by a large number of people with a history of alcohol ingestion, the apparent overall risk appears low. A systematic review of acetaminophen use in alcoholic subjects concluded that there was little credible evidence implicating therapeutic doses of acetaminophen as a cause for fulminant hepatotoxicity in alcoholics [2]. Nevertheless, the United States Food and Drug Administration (FDA) requires that the labels of non-prescription analgesic drugs, including acetaminophen products, warn patients who consume more than three alcoholic drinks daily to discuss the use of acetaminophen with their doctor.

The principal determinant of acetaminophen-induced liver injury is believed to be the reactive metabolite, N-acetyl-p-benzoquinoneimine (NAPQI), produced primarily by CYP2E1-mediated oxidation of acetaminophen. The primary defense against the toxic actions of this metabolite is the hepatic store of reduced glutathione (GSH). Plasma GSH concentrations are thought to provide a reasonable surrogate assessment of hepatic GSH stores [3]. Published data suggest that that the plasma GSH concentration is decreased in alcoholic subjects [4], particularly in those ingesting acetaminophen [5].

Previous research has shown that a 2-day course of acetaminophen in subjects with chronic alcoholism did not cause changes in aspartate aminotransferase (AST), alanine aminotransferase (ALT), bilirubin or international normalized ratio (INR). It has been proposed that the 2-day course of treatment was of insufficient duration to induce hepatic injury [6-9]. The purpose of this study was twofold: (1) to determine if a 3-day course of acetaminophen dose (4.0 g/day) affects the serum ALT, AST or INR, and (2) to determine if the plasma concentration of reduced glutathione is affected by this acetaminophen dosing regimen.

## Methods

A randomized double-blind placebo-controlled trial of alcoholic subjects 18 years or older, admitted to the Denver Health Comprehensive Addictions Rehabilitation and Evaluation Services (CARES) in Denver, CO, USA or Recovery Centers of King County (RCKC) in Seattle, WA, USA from January 2002 through July 2004 was conducted. The study consisted of 3 consecutive days of study drug dosing followed by 2 days of observation with continuous monitoring for the 5-day period.

Participants were eligible for enrollment if they had a detectable breath ethanol concentration at admission to the detoxification facility. Exclusion criteria included a baseline serum AST or ALT above 200 IU/L, baseline INR above 1.5, baseline serum acetaminophen concentration above 20 mcg/mL, a history of ingesting more than 4 g/day of acetaminophen for any of the 4 days preceding enrollment, a history of allergy to acetaminophen, enrollment in any other trial within the preceding 3 months, or a positive serum pregnancy test.

A structured history and physical was used to assess medication and alcohol use as well as nutritional status. Formal screening for alcoholism included the CAGE questionnaire and the Brief Michigan Alcoholism Screening Test (MAST) [10-13]. Nonprescription and prescription medication use was documented with specific questioning about ingestion of substances that bind to, induce or inhibit CYP2E1 (e.g. isoniazid, chlorzoxazone, disulfiram). Study investigators clinically classified each participant into one of the following categories: normal nutritional status, mild malnutrition, or severe malnutrition [14]. Body mass index (BMI kg/m^2^) was calculated.

The study was approved by the institutional review boards governing the institutions involved, namely the Colorado Multiple Institutional Review Board (COMIRB) for CARES and the Western Institutional Review Board (WIRB) for RCKC. Written informed consent was obtained from all subjects after they were documented to have nondetectable breath ethanol levels. Each subject received $10.00 per day.

### Assignment

Each eligible participant was randomized to receive either acetaminophen or placebo at a 2:1 ratio. An investigator not involved with participant management generated the randomization codes for each site using statistical software (StatMate version 1.011; GraphPad Software Inc, San Diego, CA, USA). Group assignments were blinded for participants, staff and investigators.

### Masking

Acetaminophen was purchased from a retail store (Extra Strength Tylenol^® ^tablets, 500 mg per caplet; McNeil Consumer and Specialty Pharmaceuticals, Fort Washington, PA, USA). Placebo capsules contained granulated sugar. To maintain blinding, placebo and acetaminophen doses were placed in identical pill casings and stored in uniquely coded individual bags by study personnel not involved in evaluation or management of subjects.

### Participant flow and follow up

Baseline blood specimens were drawn in the morning prior to administration of study medication and each morning of study days 2 through 5 in order to avoid the effects of diurnal variation. Baseline testing included serum acetaminophen level, complete blood cell count (CBC), serum electrolytes with renal function tests, hepatic panel (AST, ALT, and bilirubin), INR, γ-glutamyl transferase (GGT) and, in female subjects, serum β-human chorionic gonadotropin (β-HCG). Hepatic panel and INR testing were performed on days 2 to 4 of the study. Serum acetaminophen level was repeated on day 3. Laboratory testing on day 5 was similar to baseline without GGT testing. Specimens from CARES were assayed in the clinical laboratory of Denver Health Medical Center (Denver, CO, USA). Specimens from RCKC were assayed at Dynacare Laboratories or Quest Diagnostics (Seattle, WA, USA). Breath ethanol levels were determined at initial presentation to the treatment center, usually in the evening or early morning before enrollment and prior to the first administration of study medication. Measurement of plasma GSH at baseline and again on study day 3 was completed in a subset of consecutive participants at CARES. This assay was performed at The Clinical Pharmacokinetic Laboratory of the Fred Hutchinson Cancer Research Center in Seattle, WA. A sample handling protocol was used in obtaining and processing these samples to limit pre-analytical variability.

Subjects who left the detoxification center during the study were withdrawn from the trial. Study medications, 2 × 500 mg acetaminophen capsules or 2 × placebo capsules, were dispensed by nursing staff or study investigators who directly verified and recorded the time of ingestion. At CARES, the dosing schedule was 09:00, 13:00, 17:00 and 23:00. At RCKC, the dosing schedule was 08:30, 12:30, 16:30 and 20:30 for the first eight participants and then 08:00, 12:00, 16:00, and 20:00 for the remaining participants.

The study physician was notified of any subject who developed an ALT or AST level > 200 IU/L. The blind was broken for these subjects to assist the study physician in making treatment decisions. A total of 10 participants (8 acetaminophen, 2 placebo) were unblinded in this manner. No participant suffered acetaminophen-related liver injury.

### Analysis

The primary outcome of interest was a change in the serum ALT activity or the INR. Secondary outcomes were the serum bilirubin and the plasma glutathione concentrations. Demographic and clinical laboratory data for all subjects in the two study groups were compared using a statistical software package (SPSS Inc, Chicago IL, USA). Chi-Square or exact tests were used to compare categorical data such as gender and ethnicity, while an unpaired Student *t *test or analysis of variance (ANOVA) was used to compare continuous numerical data such as age and most laboratory findings. Repeated measures analysis of variance (ANOVA) was used to compare continuous numerical data (i.e. ALT, AST) within groups from greater than three time points. In all analyses, a two-sided p value < 0.05 was considered significant.

## Results

**Figure 1:**
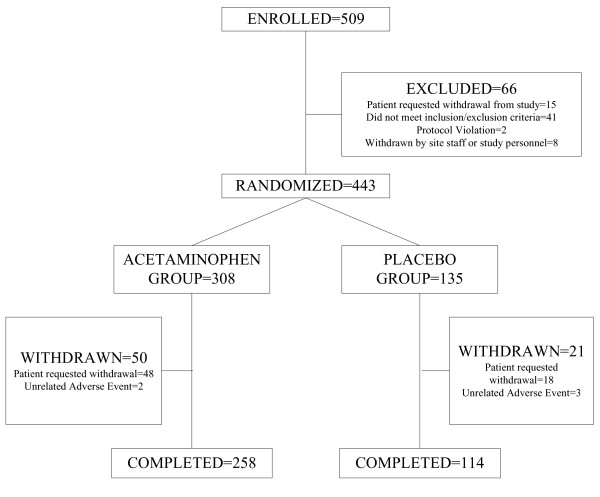
Disposition of participants.

Of the 509 subjects screened for eligibility, 66 were excluded prior to receiving study medication (Figure 1). Of these 66 excluded subjects, 24 were due to baseline serum AST or ALT > 200 IU/L, 16 presented to the facility with nondetectable breath ethanol, 15 requested to be withdrawn, 1 had a baseline INR greater than 1.5, 2 were removed due to protocol violations, and 8 because the nursing staff determined that the facility could not accommodate these subjects, usually for behavioral reasons.

The remaining 443 participants were randomized: 308 subjects were assigned to receive acetaminophen and 135 subjects were assigned to receive placebo. After randomization, 50 subjects withdrew from the acetaminophen group and 21 from the placebo group (Figure 1). Five participants withdrew due to adverse events unrelated to the study medication: 2 in the acetaminophen group (both from alcohol withdrawal symptoms) and 3 in the placebo group (1 from frostbite, 1 from alcohol withdrawal symptoms, 1 from abdominal pain). The remaining subjects (48 in the acetaminophen group, 18 in the placebo group) withdrew from the study because they did not want to remain in the treatment facility. Withdrawal rates were 16% for both the acetaminophen and placebo groups (p > 0.05).

Study groups were of similar demographic and baseline characteristics, including baseline serum AST, ALT, INR, and bilirubin (Table 1). Minority ethnic groups were well represented. More than 80% of study participants reported their most recent drinking episode lasting more than 1 week (Table 1).

Analyses of ALT and AST were highly concordant, so only ALT results are shown. The mean baseline serum ALT of the acetaminophen group was 49 ± 38 IU/L and 47 ± 37 IU/L in the placebo group. Both the acetaminophen and placebo groups experienced statistically significant variation in the serum ALT during their course; however, there was no difference between the two groups at any point (Table 2). For both groups, the highest mean ALT occurred on day 5. The study had 95% probability of detecting a mean difference in the serum ALT of 15 IU/L. There was no significant change in mean INR, total bilirubin, or any other laboratory analysis between the study groups throughout the study (p > 0.05).

**Figure 2:**
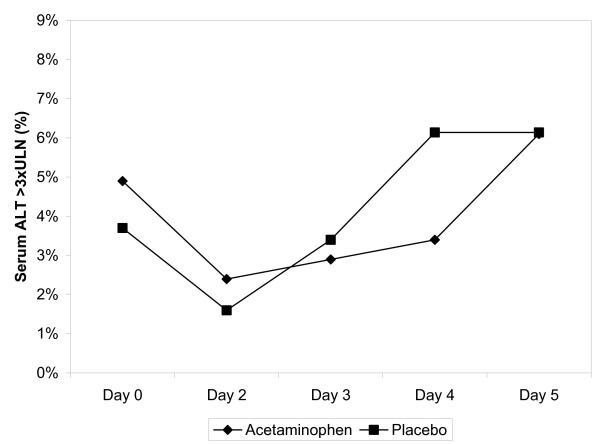
Incidence of alanine aminotransferase (ALT) measures greater than three times upper limit of normal throughout study by treatment group.

A series of post-hoc analyses were performed to explore the possibility that a subgroup of participants could have had a different response to acetaminophen administration (i.e. that a susceptible subpopulation exists). None of the analyses indicated a statistical difference between the acetaminophen and placebo groups, although some analyses had few subjects and limited power to detect a difference. The ALT stayed the same or decreased from baseline in 31% of subjects in the placebo group and 36% of subjects in the acetaminophen group. The ALT increased above baseline in 66% of participants with a maximum increase of 1–254 IU/L in the acetaminophen group and 1–158 IU/L in the placebo group. A total of 32 participants (24 acetaminophen, 8 placebo) developed an ALT greater than three times the upper limit of normal (ULN) at some point during the course of the study (Figure 2). A total of 11 subjects developed an ALT greater than 200 IU/L at some time during the study (9 acetaminophen, 2 placebo). The maximum ALT and AST measures were 312 and 448 IU/L, respectively. One subject developed coagulopathy; this subject was in the placebo group and had an INR of 1.8 on day 3. A bilirubin that exceeded the upper limit of normal at any time was found in 20 (6.4%) in the acetaminophen group and 6 (4.4%) in the placebo group.

**Figure 3:**
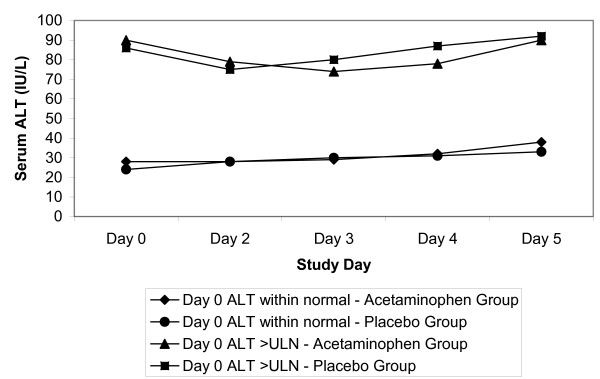
Serum alanine aminotransferase (ALT) stratified by baseline ALT value.

There were 156 participants (106 acetaminophen, 50 placebo) that entered the study with a serum ALT above the ULN. The ALT group means of those with a baseline ALT greater than the ULN were consistently two to three times higher throughout the trial, regardless of group assignment (Figure 3).

**Figure 4:**
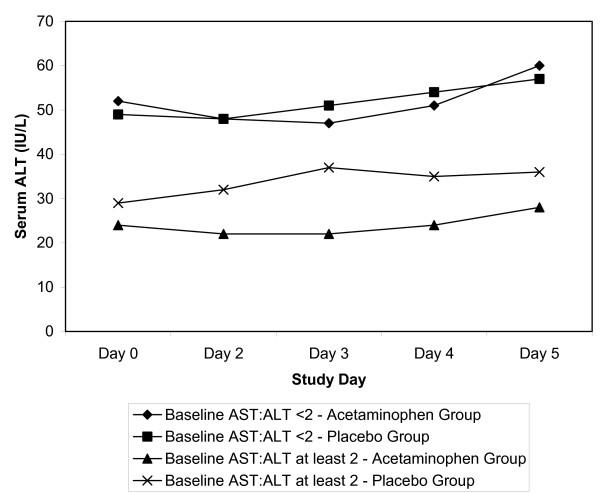
Serum alanine aminotransferase (ALT) in patients with alcoholic liver disease.

To assess the effect of acetaminophen administration on participants fulfilling a case definition of alcoholic liver disease, a post-hoc analysis was performed using the 44 subjects in whom the baseline AST/ALT ratio was 2.0 or greater [15]. The mean ALT of the group without evidence of alcoholic liver disease was significantly higher throughout the trial than the group with evidence of alcoholic liver disease; however, the acetaminophen and placebo groups were not statistically different (Figure 4). The peak ALT measure of these 44 subjects was 75 IU/L compared to 312 IU/L for the entire study sample. No subject with an AST to ALT ratio of 2.0 or greater developed an ALT more than three times the ULN or reported any study-related adverse events.

Plasma reduced glutathione concentration was determined at baseline and on day 3 in a subset of 79 consecutive participants (56 acetaminophen, 23 placebo). Plasma glutathione increased from 2.17 ± 0.97 to 2.27 ± 0.85 μM in the acetaminophen group and from 1.90 ± 0.68 to 2.02 ± 0.74 μM in the placebo group. There was no difference between treatment or placebo groups (p > 0.05). The correlation between plasma GSH concentration and ALT, AST or INR was not significant (p > 0.05).

The role of nutritional status was explored. Neither body mass index nor any degree of malnutrition correlated with increases in ALT. There were eight serious adverse events experienced by five patients: one infection with methicillin resistant *staphylococcus*, one abdominal pain, three patients who developed delirium tremens, two patients developed confusion and one patient developed ataxia. These events were deemed serious because they resulted in hospitalization of the patient. One event was possibly related to the study medication. This patient experienced abdominal pain after receiving three doses of placebo.

## Discussion

The administration of acetaminophen 4 g/day for 3 consecutive days to newly-abstinent chronic alcoholic subjects did not result in a change in serum ALT, AST, bilirubin, or INR. Our study had 95% power to detect a 15 IU/L change in AST or ALT, a smaller change than is typically used by common definitions of liver injury [16].

This study was designed to safely maximize the opportunity to detect a potential alcohol-acetaminophen interaction. Participants had been drinking alcohol for a prolonged period and abruptly discontinued alcohol intake upon presentation to the detoxification facility. This presentation reproduces the conditions commonly cited in reports of acetaminophen injury associated with therapeutic doses in the medical literature: namely, induction of CYP2E1 and concomitant depletion of glutathione [17-20].

Hepatic injury from acetaminophen is caused by the production of a reactive metabolite N-acetyl-p-benzoquinoneimine (NAPQI). Only a small percentage of acetaminophen is converted by CYP2E1 to NAPQI, which is normally detoxified by hepatic glutathione (GSH). If GSH stores are depleted, NAPQI can bind to hepatocellular components resulting in liver injury. Conditions which can increase NAPQI production, such as induction of CYP2E1 by alcohol, or conditions that decrease GSH stores, such as malnutrition, are postulated to increase the risk of acetaminophen-induced hepatic injury [5,17,19-24]. Human studies have demonstrated that chronic alcohol exposure can increase CYP2E1 activity, up to approximately twice baseline [12]. Increased CYP2E1 activity begins immediately upon exposure to ethanol [21]. Following cessation of chronic alcohol exposure, human studies have also demonstrated that CYP2E1 induction is transient, lasting at most a few days [25]. Animal and human data suggest that different mechanisms can contribute, to different degrees and at different ethanol concentrations, to increasing CYP2E1 activity [8,9,26,27].

The alcohol-induced induction of CYP2E1 wanes following alcohol abstinence with a half-life of approximately 2.5 days and CYP2E1 activity reaching normal in 3 to 8 days [12,25,28]. Our subjects were treated with acetaminophen in a period of probable CYP2E1 induction and potentially reduced plasma glutathione concentration, recreating the conditions postulated to allow liver injury in acetaminophen-treated patients who ingest alcohol.

As it is possible that subjects who did not develop transaminase abnormalities secondary to acetaminophen could obscure a potential detrimental effect in a small sub-group, several post-hoc analyses were performed in an attempt to identify trends that would guide further study of alcoholic patients. The analyses included the subgroup of participants that presented with an initially elevated ALT or AST, participants meeting a common definition of alcoholic hepatitis, the relationship of body mass index to change of ALT and several others. These analyses are limited by the reduction in statistical power created by the reduced number of subjects present in a subgroup analysis. However, none of the analyses identified a group that responded to acetaminophen ingestion with an increase in serum ALT or AST, bilirubin concentration or INR.

The medical literature contains few prospective studies involving administration of acetaminophen to newly-abstinent alcoholic patients. Lauterburg found that plasma glutathione concentration was decreased in eight newly-abstinent alcoholic subjects and decreased further following a single supratherapeutic dose of acetaminophen (2 g) [5]. As shown by the increase in glutathione in both experimental groups, our results suggest that the alcoholic patient has blood concentrations of glutathione that increase when alcohol ingestion is terminated. In contrast to the Lauterburg study, our subjects group receiving acetaminophen 1 g four times daily did not develop a decrease in the plasma concentration of glutathione.

Our results differ from Watkins et al, who reported that 31% to 44% of healthy young acetaminophen treated patients experienced an ALT greater than three times the upper limit of the normal range (3 × ULN) during treatment with the acetaminophen, 4 g/day for 14 days [29]. In contrast only 6% of our patients developed an ALT greater than 3 × ULN after 3 days of treatment and the same rate was observed in the placebo group. There are several potential explanations for this difference. First, the subject groups are considerably different: young normal subjects vs older alcoholic subjects. Alcoholic subjects repeatedly insult their liver, which might decrease the response to another stimulus. Second, although daily ALT values were not provided in the Watkins manuscript, the author stated that "Elevation of ALT to more than three times the ULN (120 U/L) was not observed prior to day 3 in any of the treatment groups." Similarly, our results indicate that the proportion of patients with an ALT greater than 3 × ULN (some patients started with ALT > ULN) decreased on day 2 and then increased slightly on days 3, 4 and 5 (Figure 2). Although the increase in ALT also occurred in the control group, we cannot exclude that the serum ALT of our patients would not have followed a course similar to those reported in Watkins. Finally, previous randomized trials established previously that the serum aminotransferase activity increases in some patients during treatment with acetaminophen, however, none of those studies determined ALT as frequently (daily) or as early in the course as Watkins [30]. The meaning of asymptomatic increases in serum ALT is unknown. Further research with administration for longer periods in alcoholic patients is needed to answer these questions.

Previous research by our group involving administration of acetaminophen to alcoholic subjects for 2 days supports the findings of the current study [31,32]. Therapeutic acetaminophen dosing in a total of 132 subjects showed no effect on serum ALT, AST, bilirubin or INR. Recently, Bartels et al studied the administration of acetaminophen for 4 days. He also found no difference in serum glutathione S-transferase (GST), an indicator of hepatocyte enzyme release similar to ALT and AST [13]. Although they are few, all prospective studies in alcoholic subjects to date have failed to find liver injury associated with therapeutic doses of acetaminophen.

There are limitations to the generalizability of our results. We only studied the maximal recommended daily dose of acetaminophen (4.0 g/day), so our data should not be extrapolated to either intentional or unintentional overdoses. Larson et al reported on patients entered into the Acute Liver Failure registry [33]. Among patients with liver injury thought to be acetaminophen-related, referred to a liver service participating in the registry and fulfilling the diagnosis of acute liver failure, 51% reported therapeutic intent and 63% reported use of an acetaminophen-opioid combination drug. Most patients ingested an overdosage of acetaminophen with a mean dosage of 7.5 g/day (range 1–78 g/day). It is crucial to distinguish between ingestion of a true therapeutic dose and ingestion of an overdose despite therapeutic intent.

Our results do not apply to all alcoholic patients. By including subjects with higher aminotransferase levels than previously studied, we were able to enroll more than 95% of alcoholic volunteers screened. However, we did not study patients with decompensated alcoholic liver disease. Our results might therefore not apply to patients with liver disease severe enough to impair hepatic synthetic function.

We evaluated a 3-day course of acetaminophen therapy. As cytochrome induction wanes substantially in the first 2 days, an alcoholic patient would be expected to be at highest risk during the first day following cessation of alcohol intake. We did not study alcoholic patients that continued to ingest alcohol concurrently with acetaminophen. However, acute ingestion of alcohol inhibits rather than increases NAPQI formation, so it is unlikely that acute alcohol consumption would increase the susceptibility of alcoholic patients to liver injury [2,21,24,34]. The metabolism of acetaminophen produces a protein adduct of acetaminophen composed of a cysteine residue covalently bound to acetaminophen. In the future, this assay could allow quantitation of the amount of acetaminophen actually metabolized by a patient and allow stratification and analysis of the risk of hepatotoxicity in patients receiving acetaminophen [35,36].

Claims of a potential acetaminophen-alcohol interaction have prompted some healthcare providers to recommend a decreased dose or complete avoidance of acetaminophen for patients who drink alcohol. If patients who drink alcohol are instructed to avoid acetaminophen altogether, they will likely use other OTC analgesics such as aspirin or other nonsteroidal anti-inflammatory medications (NSAIDs). NSAIDs and aspirin cause significant morbidity and mortality at or near the therapeutic dosage. Precise data are not available, but it is estimated that 100,000 Americans are hospitalized and 16,000 patients die each year in the United States from gastrointestinal hemorrhage, ulcers and perforations secondary to NSAIDs and aspirin [15,20,35,37]. The FDA has recognized the significance of this risk and since 1998 has mandated that OTC NSAIDs such as ibuprofen and naproxen sodium carry an alcohol warning.

The alcohol warning on all common OTC analgesics advises people who consume three or more alcoholic drinks every day to consult a doctor before using these drugs. Healthcare providers are commonly faced with the question of which OTC analgesic is the safest for patients who occasionally drink alcohol as well as for those patients who are suspected or confirmed alcoholics. The answer to this question requires an assessment of the risks of each analgesic. A recent study suggested that NSAID treatment produces a tenfold increase in risk of gastrointestinal bleeding for patients within 1 week of initiating therapy [38]. Although we did not directly compare the safety of NSAIDS to acetaminophen, we were unable to find any evidence of liver injury in "high risk" subjects ingesting acetaminophen. Acetaminophen is present in many OTC products. Inadvertent concurrent use of these products could exceed the maximum recommended dosage of 4 g/day. The relative susceptibility of alcoholic patients and other patients in doses above 4 g/day is unclear.

## Conclusion

Overall, treatment of pain or fever for 3 days with acetaminophen appears safe in newly-abstinent alcoholic patients, such as those presenting for acute medical care. This study and other prospective data suggest that short-term maximal therapeutic acetaminophen dosing does not cause liver injury in alcoholics.

## List of Abbreviations

ALT, alanine aminotransferase; ANOVA, analysis of variance; AST, aspartate aminotransferase; β-HCG, β-human chorionic gonadotropin; BMI, Body Mass Index; CARES, Comprehensive Addictions Rehabilitation and Evaluation Services; CBC, complete blood count; CYP2E1, cytochrome P4502E1; FDA, Food and Drug Administration; GGT, gamma glutamyl transferase; GSH, glutathione reduced form; GST, glutathione-S-transferase; INR, International normalized ratio; IU, international units; L, liter; NAPQI, N-acetyl-p-benzoquinoneimine; NSAIDS, Nonsteroidal anti-inflammatory drugs; OTC, over the counter; RCKC, Recovery Centers of King County; ULN, upper limit of normal.

## Competing interests

At the time of this study, EKK had no competing interests. However, in 2006, EKK became an employee of McNeil Consumer Healthcare, the financial sponsor of this study. JLG, GMB, KH and RCD are employees of Denver Health Rocky Mountain Poison & Drug Center (Denver Health and Hospital Authority), a non-profit governmental facility that provides poison and drug information to various entities under contract. These entities include the states of Colorado, Montana, Idaho, Nevada and Hawaii. Business clients include McNeil Consumer & Specialty Pharmaceuticals, the manufacturer of Motrin (ibuprofen) and Tylenol (acetaminophen). McNeil provided financial support for this study. PCK, RBP and JS have no competing interests.

## Authors' contributions

EK planned and conducted the trial, and wrote the manuscript. JG was the study coordinator and provided statistical design and data analysis. GB helped develop the protocol, chaired the Data Safety Management Board and reviewed the data analysis and the manuscript. PK was the RCKC site coordinator and conducted the trial at that site. RP conducted the trial and revised the manuscript. KH conducted the trial and revised the manuscript. JS planned the trial, performed glutathione level tests, and revised the manuscript. RD conceived and planned the trial, obtained research funding and revised the manuscript.

## Pre-publication history

The pre-publication history for this paper can be accessed here:



## Figures and Tables

**Table 1:** Baseline characteristics of patients in acetaminophen and placebo groups

	Acetaminophen group (n = 308)	Placebo group (n = 135)	p-value*
Age:			
Mean ± SD	43 ± 9	44 ± 8	0.399
Range	18–72	20–65	
Male (%)	284 (92)	128 (95)	0.322
Ethnic Origin (%):			
Caucasian	156 (51)	68 (50)	0.830
Hispanic	45 (15)	22 (16)	
African Descent	49 (16)	16 (12)	
Native American	43 (14)	23 (17)	
Other	15 (5)	6 (4)	
Body Mass Index:			
Mean ± SD	25 ± 5	25 ± 4	
Range	15–55	17–39	
Breath alcohol level at presentation to detoxification site:			0.711
Mean ± SD	0.161 ± 0.093	0.157 ± 0.099	
Range	0.001–0.710	0.001–0.550	
Self-reported alcoholic (%)	301 (98)	133 (99)	0.729
Duration of most recent drinking binge:			
< 1 week	56 (18)	22 (16)	0.193
1–4 weeks	98 (32)	36 (27)	
> 1 month	154 (50)	76 (56)	
Not reported	0 (0)	1 (1)	
CAGE (no. of yes answers; mean ± SD)	3.3 ± 0.9	3.3 ± 0.9	0.645
Alanine aminotransferase:			
mg/dL	49 ± 38	47 ± 37	0.398
Range	4–189	8–175	
Aspartate aminotransferase:			
IU/L	55 ± 39	55 ± 38	0.650
Range	6–198	12–194	
International normalized ratio:			0.652
INR	0.96 ± 0.09	0.96 ± 0.09	
Range	0.79–1.40	0.80–1.30	
Albumin:			0.589
g/dl	3.94 ± 0.45	3.93 ± 0.37	
Range	2.1–4.8	2.8–4.0	
Total bilirubin:			
mg/dL	0.74 ± 0.53	0.68 ± 0.45	0.286
Range	0.1–4.1	0.1–2.8	
Gamma glutamyl transferase:			
IU/L	161 ± 308	168 ± 274	0.799
Range	9.0–3492	12.0–1893	

*Standardized values based upon upper limit of normal were used to calculate p-values.

Normal ranges for Denver and Seattle sites are as follows. Alanine aminotransferase: Denver, ALT 5–40 mg/dL male, 5–36 mg/dL female; Seattle, 0–50 IU/L and 2–60 IU/L male, 2–40 IU/L female. Aspartate aminotransferases: Denver, 9–36 IU/L; Seattle: 0–50 IU/L and 2–50 IU/L male, 2–35 IU/L female (both). International normalized ratio: Denver, 0.82–1.17; Seattle, 0.9–1.1. Total bilirubin: Denver, 0.2–1.2 mg/dL; Seattle: 0.1–1.2 mg/dL and 0.2–1.5 mg/dL male, 0.2–1.3 mg/dl female. Gamma glutamyl transferase: Denver, 6–50 IU/L; Seattle: 5–105 IU/L male, 2–98 IU/L female and 2–80 IU/L male, 2–60 IU/L female. Albumin: Denver, 3.4–5.5 g/dl; Seattle, 3.3–5.0 and 3.5–4.9 g/dl

**Table 2:** Serum alanine aminotransferase (ALT) measures (intent to treat analysis)

	Serum ALT (IU/L)		
	
	Acetaminophen group	Placebo Group	p-value*
Day 0:			
Mean ± SD	49 ± 38	47 ± 37	0.398
Range	4–189	8–175	
n	308	135	
Day 2:			
Mean ± SD	45 ± 38	46 ± 34	0.949
Range	7–292	6–174	
n	287	124	
Day 3:			
Mean ± SD	45 ± 36	50 ± 38	0.245
Range	7–230	6–242	
n	274	118	
Day 4:			
Mean ± SD	48 ± 39	52 ± 43	0.411
Range	6–264	8–288	
n	267	115	
Day 5:			
Mean ± SD	57 ± 45	55 ± 48	0.706
Range	7–312	7–282	
n	261	114	

*Standardized values based upon upper limit of normal were used to calculate p-values.

Normal ranges for CARES: ALT 5–40 IU/L male and 5–36 IU/L female. Two different labs were used by RCKC, normal ranges: ALT 0–50 IU/L and 2–60 IU/L male, 2–40 IU/L female.

## References

[B1] Kaufman DW, Kelly JP, Rosenberg L, Anderson TE, Mitchell AA (2002). Recent patterns of medication use in the ambulatory adult population of the United States: the Slone survey. JAMA.

[B2] Dart RC, Kuffner EK, Rumack BH (2000). Treatment of pain or fever with paracetamol (acetaminophen) in the alcoholic patient: a systematic review. Am J Ther.

[B3] Barbaro G, DiLorenzo G, Soldini M (1996). Hepatic glutathione deficiency in chronic hepatitis C: quantitative evaluation in patients who are HIV positive and HIV negative and correlations with plasmatic and lymphocytic concentrations and with the activity of the liver disease. Am J Gastroenterol.

[B4] Shaw S, Rubin K, Lieber CS (1983). Depressed hepatic glutathione and increased diene conjugates in alcoholic liver disease. Digest Dis Sci.

[B5] Lauterburg BH, Velez ME (1988). Glutathione deficiency in alcoholics: risk factor for paracetamol hepatotoxicity. Gut.

[B6] Soll AH, Sees KL (2002). Is acetaminophen really safe in alcoholic patients?. Arch Intern Med.

[B7] Oviedo J, Wolfe MM (2002). Alcohol, acetaminophen, and toxic effects on the liver. Arch Intern Med.

[B8] Badger TM, Huang J, Ronis M, Lumpkin CK (1993). Induction of cytochrome P450 2E1 during chronic ethanol exposure occurs via transcription of the CYP 2E1 gene when blood alcohol concentrations are high. Biochem Biphys Res Commun.

[B9] Takahashi T, Lasker JM, Rosman AS, Lieber CS (1193). Induction of cytochrome P4502E1 in the human liver by ethanol is caused by a corresponding increase in encoding messenger RNA. Hepatology.

[B10] Mayfield D, McLeod G, Hall P (1974). The CAGE questionnaire: validation of a new alcoholism screening instrument. Am J Psychiatry.

[B11] Kristenson H, Trell E (1982). Indicators of alcohol consumption: comparisons between a questionnaire (Mm-MAST), interviews and serum gamma-glutamyl transferase (GGT) in a health survey of middle-aged males. Br J Addict.

[B12] Banda PW, Quart BD (1982). The effect of mild alcohol consumption on the metabolism of acetaminophen in man. Res Comm Chem Pathol Pharmacol.

[B13] Bartels SA, Crosby DL, Richard JI, Sivilotti MLA (2005). Does maximal therapeutic dosing of acetaminophen perturb hepatic biomarkers in recently abstinent alcoholics?. Clin Toxicol.

[B14] Baker JP, Detsky AS, Wesson DE, Wolman SL, Stewart S, Whitewell J, Langer B, Jeejeebhoy KN (1982). Nutritional assessment: a comparison of clinical judgment and objective measurements. N Engl J Med.

[B15] Pratt DS, Kaplan MM (2000). Evaluation of abnormal liver-enzyme results in asymptomatic patients. N Engl J Med.

[B16] Navarro VJ, Senior JR (2006). Drug-related hepatotoxicity. N Engl J Med.

[B17] Seeff LB, Cuccherini BA, Zimmerman HJ, Adler E, Benjamin SB (1986). Acetaminophen hepatotoxicity in alcoholics. A therapeutic misadventure. Ann Intern Med.

[B18] Black M, Raucy J (1986). Acetaminophen, alcohol, and cytochrome P-450. Ann Intern Med.

[B19] Zimmerman HJ, Maddrey WC (1995). Acetaminophen (paracetamol) hepatotoxicity with regular intake of alcohol: analysis of instances of therapeutic misadventure. Hepatology.

[B20] Peura DA, Lanza FL, Gostout CJ (1997). The American College of Gastroenterology Bleeding Registry: preliminary findings. Am J Gastroenterol.

[B21] Slattery JT, Nelson SD, Thummel KE (1996). The complex interaction between ethanol and acetaminophen. Clin Pharmacol Ther.

[B22] Nelson SD (1990). Molecular mechanisms of the hepatotoxicity caused by acetaminophen. Semin Liver Dis.

[B23] Bray GP, Mowat C, Muir DF, Tredger JM, Williams R (1991). The effect of chronic alcohol intake on prognosis and outcome in paracetamol overdose. Hum Exper Toxicol.

[B24] Critchley JA, Dyson EH, Scott AW, Jarvie DR, Prescott LF (1983). Is there a place for cimetidine or ethanol in the treatment of paracetamol poisoning?. Lancet.

[B25] Lucas D, Menez C, Girre C (1995). Decrease in cytochrome P4502E1 as assessed by the rate of chlorzoxazone hydroxylation in alcoholics during the withdrawal phase. Alcohol Clin Exp Res.

[B26] Ronis MJ, Huang J, Crouch J, Mercado C, Irby D, Valentine CR, Lumpkin CK, Ingelman-Sundberg M, Badger TM (1993). Cytochrome P450 2E1 induction during chronic alcohol exposure occurs by a two-step mechanism associated with blood alcohol concentrations in rats. J Pharmacol Exp Ther.

[B27] Badger TM, Ronis MJ, Ingelman-Sundberg M, Hakkak R (1993). Pulsatile blood alcohol and CYP2E1 induction during chronic alcohol infusions in rats. Alcohol.

[B28] Oneta CM, Lieber CS, Li J, Ruttimann S, Schmid B, Lattmann J, Rosman AS, Seitz HK (2002). Dynamics of cytochrome P4502E1 activity in man: induction by ethanol and disappearance during withdrawal phase. J Hepatol.

[B29] Watkins PB, Kaplowitz N, Slattery JT, Colonese CR, Colucci SV, Stewart PW, Harris SC (2006). Aminotransferase elevations in healthy adults receiving 4 grams of acetaminophen daily. JAMA.

[B30] Bradley JD, Brandt KD, Katz BP, Kalasinski LA, Ryan SI (1991). Comparison of an antiinflammatory dose of ibuprofen, an analgesic dose of ibuprofen, and acetaminophen in the treatment of patients with osteoarthritis of the knee. N Engl J Med.

[B31] Kuffner EK, Bogdan GM, Dart RC (1997). Evaluation of hepatotoxicity in alcoholics from therapeutic dosing of acetaminophen. J Toxicol Clin Toxicol.

[B32] Kuffner EK, Dart RC, Bogdan GM, Hill RE, Casper E, Darton L (2001). Effect of maximal daily doses of acetaminophen on the liver of alcoholic patients: a randomized, double-blind, placebo-controlled trial. Arch Intern Med.

[B33] Larson AM, Polson J, Fontana RJ (2005). Acetaminophen-induced acute liver failure: results of a United States multicenter, prospective study. Hepatology.

[B34] Banda PW, Quart BD (1982). The effect of mild alcohol consumption on the metabolism of acetaminophen in man. Res Comm Chem Pathol Pharmacol.

[B35] James LP, Alonso EM, Hynan LS, Hinson JA, Davern TJ, Lee WM, Squires RH, Pediatric Acute Liver Failure Network (2006). Detection of acetaminophen protein adducts in children with acute liver failure of indeterminate cause. Pediatrics.

[B36] Davern TJ, James LP, Hinson JA, Polson J, Larson AM, Fontana RJ, Lalani E, Munoz S, Shakil AO, Lee WM, Acute Liver Failure Study Group (2006). Measurement of serum acetaminophen-protein adducts in patients with acute liver failure. Gastroenterology.

[B37] Blot WJ, McLaughlin JK (2000). Over the counter non-steroidal anti-inflammatory drugs and risk of gastrointestinal bleeding. J Epidemiol Biostat.

[B38] Lewis SC, Langman MJ, Laporte JR, Matthews JN, Rawlins MD, Wiholm BE (2002). Dose-response relationships between individual nonaspirin nonsteroidal anti-inflammatory drugs (NANSAIDs) and serious upper gastrointestinal bleeding: a meta-analysis based on individual patient data. Br J Clin Pharmacol.

